# Anxiety and depression in Nepal: prevalence, comorbidity and associations

**DOI:** 10.1186/s12888-016-0810-0

**Published:** 2016-04-14

**Authors:** Ajay Risal, Kedar Manandhar, Mattias Linde, Timothy J. Steiner, Are Holen

**Affiliations:** Department of Neuroscience, Norwegian University of Science and Technology (NTNU), Trondheim, NO 7491 Norway; Dhulikhel Hospital, Kathmandu University Hospital, Dhulikhel, Kavre Nepal; Norwegian Advisory Unit on Headaches, St Olavs University Hospital, Trondheim, Norway; Division of Brain Sciences, Imperial College London, London, UK; Pain Unit, St Olavs University Hospital, Trondheim, Norway

**Keywords:** Anxiety, Depression, Developing countries, Health policy, Mental health, Neuroticism, Public health, South Asia, Urbanization, Widowhood

## Abstract

**Background:**

Anxiety and depression are two important contributors to the global burden of disease. In many developing countries, including Nepal, their prevalences are yet to be assessed.

**Methods:**

A nationwide cross-sectional study was conducted among a representative sample of Nepalese adults aged 18–65 years (*N* = 2100), selected by multistage random cluster sampling and interviewed at home during unannounced visits. The validated questionnaires included the Hospital Anxiety and Depression Scale (HADS), to detect cases of anxiety (HADS-A), depression (HADS-D) and comorbid anxiety and depression (HADS-cAD), the Eysenck Personality Questionnaire Revised Short Form-Neuroticism (EPQRS-N), and the World Health Organization Quality of Life 8-question scale (WHOQOL-8). Logistic regression analyses were used to explore associations of caseness with four groups of variables: demographic, domicile, substance use, and behavioural and health.

**Results:**

Age- and gender-adjusted point prevalences of HADS-A, HADS-D and HADS-cAD were 16.1, 4.2 and 5.9 % respectively. In a multivariate model, HADS-A was positively associated with urban residence (AOR = 1.82; *p* < 0.001) and neuroticism (AOR = 1.32; *p* < 0.001), and negatively with alcohol consumption (AOR = 0.71; *p* = 0.041). HADS-D was positively associated with marijuana use (AOR = 3.61; *p* = 0.017) and negatively with quality of life (QoL) (AOR = 0.86; *p* < 0.001). HADS-cAD was positively associated with widowhood (AOR = 2.71; *p* = 0.002), urban residence (AOR = 2.37; *p* = 0.001), living at altitude ≥2000 m (AOR = 2.32; *p* = 0.002) and neuroticism (AOR = 1.26; *p* < 0.001), and negatively with alcohol use (AOR = 0.56; *p* = 0.026) and QoL (AOR = 0.79; *p* < 0.001).

**Conclusion:**

Depression and anxiety are important mental health conditions in Nepal, and major contributors to public ill health, being very highly prevalent, comorbid and associated with psychosocial burden. They are also linked to the unique topography, habitation and social structure of the country. High prevalence coupled with the disabling nature of these disorders establishes their health-care priority and their importance in national health policy.

## Background

Anxiety and depression embrace a range of mental conditions occurring frequently in primary health care, usually in the form of overt disorders. They are acknowledged as common mental disorders (CMDs) [1, 2] and, in terms of their ubiquity and the burdens they impose, as major disorders of the brain [3]. In the recent Global Burden of Disease Study 2013 (GBD 2013), while mental and substance use disorders collectively accounted for 21.2 % of all years of life lost to disability (YLDs) [4], depression and anxiety were ranked second and ninth highest specific causes of YLDs in both developed and developing countries. These disorders are therefore of considerable public-health importance [5] in high-income [6, 7] and low- and middle-income (LAMI) countries [8] alike. They are also highly comorbid [9].

Extrapolations from GBD 2013 data indicate that depression and anxiety are among the top ten causes of YLDs in South Asia, which includes Nepal [4]; however, no research has been undertaken to make direct national estimates in this Region. Available studies were limited to a few scattered health centres [10, 11], districts [12–14], villages [15] or cities [16], and could not comprehensively describe the prevalence of CMDs, let alone provide an account of the burdens attributable to them.

With these factors under consideration, our principal aim was to assess health-care needs by estimating the prevalences of anxiety and depression in Nepal, using a well-validated and culturally-adapted screening instrument [17]. Also, we wished to establish their degree of comorbidity, as well as their associations with sociodemographic characteristics, social behaviours and health-related factors. This would fill current knowledge gaps regarding these CMDs in the South Asian Region, and serve our overall purpose of guiding public-health policy towards better mental health in Nepal.

There were methodological issues to be considered. Most earlier South Asian studies utilized self-report scales with caseness of anxiety [10, 12] or depression [13–15] being indicated by summed scores at or above defined thresholds. Such scales are useful in areas with inadequate or unevenly distributed resources that greatly limit epidemiological surveys [18]. This was certainly true of Nepal, one of the poorest nations of the world [19]. Furthermore, its geographical and sociocultural diversities posed unique logistic and methodological difficulties which our survey had to overcome [20, 21]. We considered important the relationships between mental wellbeing and behaviours typical of life and culture in the Nepalese community [20], such as the use of alcohol and marijuana during festivals, and the common method of carrying heavy loads on the back, suspended by a tumpline around the forehead. We also considered personality traits associated with psychopathology (neuroticism), and measures of burden in the face of hardship (“life toughness”), including impairment of quality of life (QoL), since these might be pertinent. In selecting covariates for analysis of associations, we had no previous research to draw upon. However, the data were gathered in the context of a nationwide survey of headache disorders [22, 23], which incorporated a range of demographic, environmental and health variables.

## Methods

### Ethics

This study was part of a larger research project addressing the major disorders of the brain in Nepal [20], approved by the Nepal Health Research Council (NHRC), the Institutional Review Committee of Kathmandu University School of Medical Sciences, Dhulikhel Hospital, and the Regional Committee for Health and Research Ethics in Central Norway. Informed consent was given by all participants and confirmed either by signature or by fingerprint.

### Study design and sampling

This was a cross-sectional study in which unannounced household visits were made during May 2013 by trained interviewers using structured questionnaires. To obtain a representative sample of the adult general population, we used a multistage random cluster-sampling technique to select households in all three physiographic divisions of the country and, within each division, all five development regions (Far-Western, Mid-Western, Western, Central and Eastern). From each household we randomly selected one adult aged 18–65 years. This procedure has been explained in more detail elsewhere [21].

### Study instruments

#### Hospital Anxiety and Depression Scale (HADS)

We used a validated Nepali translation of HADS [17] to estimate the prevalence of anxiety and depression. HADS consists of 14 items in two subscales: HADS-Anxiety and HADS-Depression, each of seven items. In relation to each item, participants report their subjective experience during the preceding week, which is rated 0–3 (3 indicating maximum symptom severity). The sum of each subscale has a potential range of 0–21. As recommended in the original English version [24], and validated in the Nepali translation [21], we used a threshold of 11 on the respective subscale to indicate caseness for anxiety or depression.

#### Eysenck personality questionnaire revised short form-neuroticism (EPQRS-N)

We used a validated Nepali translation of EPQRS-N [25] to assess the degree of neuroticism [26] in the survey participants. EPQRS-N has 12 items, each a question with response options “No” (scored 0) and “Yes” (scored 1). The sum of responses has a potential range of 0–12, higher values indicating more neuroticism.

#### World Health Organization Quality-of-Life 8-question scale (WHOQOL-8)

The culturally adapted version of WHOQOL-8 [27] was also used. This instrument consists of eight questions addressing perceived aspects of a person’s QoL: satisfaction with health and with oneself, the ability to perform daily activities, personal relations and living conditions, the sense of subjective adequacy of available resources, and the sense of having enough energy and means to meet one’s needs. Each question has five response options on a Likert scale, and is scored from 1 (worst) to 5 (best); the summed score has the potential range of 8–40. Higher total scores indicate better QoL.

#### HARDSHIP questionnaire

All these instruments were incorporated as modules into the Headache-Attributed Restriction, Disability, Social Handicap and Impaired Participation (HARDSHIP) questionnaire. This instrument was originally designed to be administered by trained lay interviewers for assessing headache disorders [28], and in our study it was translated and culturally adapted for Nepal [29]. Separate sections covered demographic characteristics (age, gender, marital status and household consumption), household location (urban or rural, and altitude), use of substances (alcohol, tobacco and marijuana), tumpline use (frequency and heaviness of load), height and weight (from which body mass index [BMI] was computed) and blood pressure (BP). Finally, we included a question on life-toughness, with five ordinal response options from “very easy” to “very tough”.

### Statistical analysis

Analyses were carried out using IBM SPSS Statistics 21.

We used caseness as defined by HADS to compute the crude prevalences of anxiety and depression. Participants scoring above the thresholds of both subscales were considered as cases of comorbid anxiety and depression (cAD). In order to examine associations, we separated HADS-cAD cases from participants scoring above the thresholds only for anxiety (HADS-A cases) or only for depression (HADS-D cases). Since HADS takes account only of very recent subjective experience (in the past week) [24], prevalence estimates approximate to point prevalences. Thus we calculated the point prevalences of anxiety, depression, HADS-A, HADS-D and HADS-cAD, reporting the estimates as percentages with 95 % confidence intervals (CIs). Our sample was under-represented by young males aged 18–34 years in comparison to the recent national population census of Nepal [21]. Hence, we adjusted our observed estimates by standardization according to the age and gender distributions of the national population [30].

We used bivariate logistic regression to identify associations with the categorical variables: demographic characteristics including age (categorized 18–25, 26–35, 36–45, 46–55, 56–65 years), habitation (urban or rural), altitude of domicile (<2000 or ≥2000 m), use of substances (yes/no for each of alcohol, tobacco and marijuana), factors related to tumpline use (frequency: never, sometimes or daily; heaviness of load [assessed subjectively]: light, moderate or heavy), life-toughness (assessed subjectively: easy, medium or tough), BP and BMI. We categorized BP as hypertensive or non-hypertensive according to the Joint National Committee on Prevention, Detection, Evaluation, and Treatment of High Blood Pressure criteria (JNC7) [31]. BMI was categorized as underweight (<18.5), normal (18.5–24.99) or overweight (≥25) [32]. We used Pearson correlation to test associations between HADS-Anxiety and HADS-Depression total scores and continuous variables: neuroticism and WHOQOL-8 scores. In view of the high number of participants relative to the number of variables in the analyses, we set the level of significance at *p* < 0.05.

Variables that showed no significant associations in bivariate analyses were excluded from multivariate analyses. The remaining variables were divided into four blocks and entered into logistic regression analyses: demographic (age and gender) in block A, domicile (habitation and altitude) in block B, substance use in block C and behavioural and health variables (tumpline use, life toughness, neuroticism and WHOQOL-8) in block D. Adjusted odds ratios (AORs) with 95 % CI for each independent variable were calculated. Age was used as a continuous variable in the multivariate computations. Prevalences of the three derived types of caseness, HADS-A, HADS-D and HADS-cAD, were the dependent variables.

## Results

The total sample included 2100 participants (males: 861 [41.0 %], females: 1239 [59.0 %], mean age 36.4 ± 12.8 years). The participation rate was 99.6 %. There were no missing data.

### Prevalence

The crude prevalence of anxiety was 22.7 % and of depression 11.7 %. The age- and gender-adjusted prevalence of HADS-A was 16.1 %, of HADS-D 4.2 % and of HADS-cAD 5.9 % (Table 1).

**Table 1:** Point prevalences (%) of each disorder according to caseness as defined (see text) (*N* = 2100)

Caseness	Point prevalence			
	*n*	%	95 % CI	Age- and gender-adjusted ^a^ (%)
Anxiety	477	22.7	20.9–24.5	-
Depression	246	11.7	10.3–13.1	-
HADS-A	340	16.2	14.6–17.8	16.1
HADS-D	109	5.2	4.2–6.1	4.2
HADS-cAD	137	6.5	5.4–7.5	5.9

^a^ Adjusted for age and gender according to the Nepal Population Report, 2011 [30]

### Associations

Anxiety caseness and depression caseness were very strongly associated (OR = 5.6 [95 % CI: 4.2–7.4]; *p* < 0.001). We arrived at this result either by taking HADS-A + HADS-cAD (all anxiety) as the independent variable and HADS-D + HADS-cAD (all depression) as the dependent, or by the reverse analysis (depression independent, anxiety dependent).

In the bivariate analysis (Table 2), HADS-A was significantly more prevalent among females (OR = 1.4). It was negatively associated with alcohol use (OR = 0.8), but more prevalent among those reporting the carrying of heavy tumpline loads (OR = 1.3) and among those who felt that life was tough (OR = 2.6). HADS-Anxiety total scores correlated positively with neuroticism (*r* = 0.57; *p* < 0.001) and negatively with WHOQOL-8 (*r* = -0.45; *p* < 0.001).

**Table 2:** Bivariate analyses of associations of caseness by HADS-A, HADS-D and HADS-cAD with categorical demographic, environmental and behavioural factors

Variable	*N*	HADS-A (*n* = 340)			HADS-D (*n* = 109)			HADS-cAD (*n* = 137)		
		Prevalence	OR	*p*	Prevalence	OR	*p*	Prevalence	OR	*p*
		*n* (%)	95 % CI		*n* (%)	95 % CI		*n* (%)	95 % CI	
Age (years)										
18–25	489	84 (17.2)	Reference	-	12 (2.5)	Reference	-	20 (4.1)	Reference	-
26–35	657	115 (17.5)	1.0 [0.8–1.4]	0.89	23 (3.5)	1.4 [0.7–2.9]	0.31	30 (4.6)	1.1 [0.6–2.0]	0.69
36–45	438	65 (14.8)	0.8 [0.6–1.2]	0.33	25 (5.7)	2.4 [1.2–4.9]	0.014	31 (7.1)	1.8 [1.0–3.2]	0.049
46–55	298	49 (16.4)	0.95 [0.6–1.4]	0.79	26 (8.7)	3.8 [1.9–7.7]	<0.001	28 (9.4)	2.4 [1.3–4.4]	0.003
56–65	218	27 (12.4)	0.7 [0.4–1.1]	0.11	23 (10.6)	4.7 [2.3–9.6]	<0.001	28 (12.8)	3.5 [1.9–6.3]	<0.001
Gender										
male	861	119 (13.8)	Reference	-	42 (4.9)	Reference	-	44 (5.5)	Reference	-
female	1239	221 (17.8)	1.4 [1.1–1.7]	0.014	67 (5.4)	1.1 [0.8–1.7]	0.59	93 (7.5)	1.5 [1.0–2.2]	0.031
Marital status										
married	1738	285 (16.4)	Reference	-	92 (5.3)	Reference	-	104 (6.0)	Reference	-
single	239	35 (14.6)	0.9 [0.6–1.3]	0.49	5 (2.1)	0.4 [0.2–0.95]	0.038	8 (3.3)	0.5 [0.3–1.1]	0.10
separated or divorced	20	4 (20)	1.3 [0.4–3.8]	0.66	2 (10)	2.0 [0.5–8.7]	0.36	1 (5.0)	0.8 [0.1–6.2]	0.85
widowed	103	16 (15.5)	0.9 [0.5–1.6]	0.82	10 (9.7)	1.9 [1.0–3.8]	0.061	24 (23.3)	4.8 [2.9–7.9]	<0.001
Household consumption (USD/year)										
≤950	822	128 (15.6)	1.0 [0.8–1.3]	0.97	54 (6.6)	1.5 [1.0–2.3]	0.065	54 (6.6)	1.1 [0.7–1.6]	0.68
950–1200	806	126 (15.6)	Reference	-	36 (4.5)	Reference	-	49 (6.1)	Reference	-
>1200	472	86 (18.2)	1.2 [0.9–1.6]	0.23	19 (4)	0.9 [0.5–1.6]	0.71	34 (7.2)	1.2 [0.8–1.9]	0.43
Habitation										
rural	1328	206 (15.5)	Reference	-	76 (5.7)	Reference	-	84 (6.3)	Reference	-
urban	772	134 (17.4)	1.1 [0.9–1.5]	0.27	33 (4.3)	0.7 [0.5–1.1]	0.15	53 (6.9)	1.1 [0.8–1.6]	0.63
Dwelling altitude										
<2000 m	1630	253 (15.5)	Reference	-	71 (4.4)	Reference	-	89 (5.5)	Reference	-
≥2000 m	470	87 (18.5)	1.2 [0.9–1.6]	0.12	38 (8.1)	1.9 [1.3–2.9]	0.002	48 (10.2)	2.0 [1.4–2.8]	<0.001
Tobacco smoking										
no	1613	265 (16.4)	Reference	-	76 (4.7)	Reference	-	91 (5.6)	Reference	-
yes	487	75 (15.4)	0.6 [0.7–1.2]	0.20	33 (6.8)	1.5 [1.0–2.2]	0.073	46 (9.4)	1.7 [1.2–2.5]	0.003
Alcohol consumption										
no	1512	260 (17.2)	Reference	-	77 (5.1)	Reference	-	106 (7.0)	Reference	-
yes	588	80 (13.6)	0.8 [0.6–0.99]	0.045	32 (5.4)	1.1 [0.7–1.6]	0.75	31 (5.6)	0.7 [0.5–1.1]	0.15
Marijuana use										
no	2065	335 (16.2)	Reference	-	104 (5)	Reference	-	134 (6.5)	Reference	-
yes	35	5 (14.3)	0.9 [0.3–2.2]	0.76	5 (14.3)	3.1 [1.2–8.3]	0.020	8 (3.6)	1.4 [0.4–4.5]	0.62
Tumpline use: frequency										
never	801	125 (15.6)	Reference	-	29 (3.6)	Reference	-	46 (5.7)	Reference	-
sometimes	532	89 (16.7)	1.1 [0.8–1.5]	0.58	28 (5.3)	1.5 [0.9–2.5]	0.15	24 (4.5)	0.8 [0.5–1.2]	0.32
daily	767	126 (16.4)	1.1 [0.8–1.4]	0.66	52 (6.8)	1.9 [1.2–3.1]	0.005	67 (8.7)	1.6 [1.1–2.3]	0.023
Tumpline use: heaviness of load										
never used	801	125 (15.6)	Reference	-	29 (3.6)	Reference	-	46 (5.7)	Reference	-
light, moderate	801	116 (14.5)	0.9 [0.7–1.2]	0.53	45 (5.6)	1.6 [1.0–2.6]	0.06	44 (5.5)	1.0 [0.6–1.5]	0.83
heavy	498	99 (19.9)	1.3 [1.0–1.8]	0.048	35 (7.0)	2.0 [1.2–3.3]	0.007	47 (9.4)	1.7 [1.1–2.6]	0.013
Life toughness										
easy	417	47 (11.3)	Reference	-	14 (3.4)	Reference	-	11 (2.6)	Reference	-
medium	1137	158 (13.9)	1.3 [0.9–1.8]	0.18	55 (4.8)	1.5 [0.8–2.7]	0.21	49 (4.3)	1.7 [0.9–3.2]	0.13
tough	546	135 (24.7)	2.6 [1.8–3.7]	<0.001	40 (7.3)	2.3 [1.2–4.2]	0.010	77 (14.1)	6.1 [3.2–11.6]	<0.001

In the multivariate analysis, the association with female gender did not survive, but the negative association with alcohol use did (AOR = 0.7 [95 % CI: 0.5–0.98]; *p* = 0.041). Additionally, a strong association emerged with urban habitation (AOR = 1.8 [95 % CI: 1.3–2.5]; *p* < 0.001). A modest negative association with daily tumpline use (AOR = 0.7 [95 % CI: 0.4–0.97]; *p* = 0.033) replaced the positive association with heavy tumpline loads. The prevalence of HADS-A was higher among high scorers on neuroticism (AOR = 1.3 [95 % CI: 1.3–1.4]; *p* < 0.001) but the negative association with WHOQOL-8, although significant, was very weak (AOR = 0.95 [95 % CI: 0.9–0.99]; *p* = 0.009).

HADS-D in the bivariate analysis (Table 2) showed a strongly ascending trend with age, the association being significant after age 35. HADS-D in this analysis was more prevalent among the high-altitude dwellers (OR = 1.9), marijuana users (OR = 3.1), daily tumpline users (OR = 1.9), those reporting carriage of heavy tumpline loads (OR = 2.0) and those reporting that their lives were tough (OR = 2.3). An association with low household consumption (OR = 1.5), a proxy for relative poverty, was not quite significant. HADS-Depression total scores correlated positively with neuroticism (*r* = 0.32; *p* < 0.001) and negatively with WHOQOL-8 (*r* = -0.50; *p* < 0.001).

Only the positive association with marijuana use (AOR = 3.6 [95 % CI: 1.2–10.5]; *p* = 0.018) and the negative association with WHOQOL-8 (AOR = 0.9 [95 % CI: 0.8–0.9]; *p* < 0.001) survived the multivariate analysis.

HADS-cAD showed a similar ascending trend with age, significant after the age of 35 years (Table 2). In the bivariate analysis, HADS-cAD was more prevalent among females (OR = 1.5), the widowed (OR = 4.8), high-altitude dwellers (OR = 2.0), tobacco users (OR = 1.7), daily tumpline users (OR = 1.6), those carrying heavy tumpline loads (OR = 1.7) and those reporting that life was tough (OR = 6.1) (Table 2).

In the multivariate analysis, the associations with widowhood (AOR = 2.7 [95 % CI: 1.5–5.1]; *p* = 0.002) and high altitude dwelling (AOR = 2.3 [95 % CI: 1.4–3.9]; *p* = 0.002) survived. Additionally, a positive association with urban dwelling (AOR = 2.4 [95 % CI: 1.4–3.9]; *p* = 0.001) and a negative association with alcohol use (AOR = 0.6 [95 % CI: 0.3–0.9]; *p* = 0.026) emerged. Multivariate analysis also uncovered a positive association of HADS-cAD with neuroticism (AOR = 1.3 [95 % CI: 1.2–1.4]; *p* < 0.001) and a negative association with WHOQOL-8 (AOR = 0.8 [95 % CI: 0.75–0.84]; *p* < 0.001).

There were no associations with BP or BMI.

## Discussion

HADS-A was more prevalent among the Nepalese than HADS-D, while the two conditions were highly comorbid with each other. HADS-cAD showed significant associations with widowhood, urban and high-altitude dwelling and neuroticism. HADS-A, like HADS-cAD, was more prevalent among urban dwellers. All three types of caseness were associated with poorer QoL. However, comorbid cases containing all elements of both anxiety and depression were associated, more than cases of HADS-A or HADS-D only, with life complications such as those of urban or high-altitude dwelling, and widowhood. We say more about these later.

Owing to the high comorbidity between anxiety and depression, psychiatric research tends to report their combined prevalence: one recent review found the collective worldwide 1-year prevalence of these disorders to be almost 20 % [5]. However, other recent global reviews have revealed prevalences separately of depression in the range 4.4–5.0 % [33] and anxiety in the range 4.8–10.9 % [34]. Our finding for HADS-D (5.2 %) was at the upper limit of the global range, but for depression (*ie*, adding those cases included among HADS-cAD), at 11.7 % (95 % CI: 10.3–13.1), it was more than double. Our finding of 16.2 % for HADS-A was already well above the global range, and for anxiety (adding those included in HADS-cAD), at 22.7 % (95 % CI: 20.9–24.5), it was again more than double. These findings are in keeping with the WHO Mental Health (WMH) survey [35], which showed anxiety disorders to be the most prevalent of all mental disorders, but suggest that, in Nepal, both depression and anxiety are excessively prevalent.

We have exercised caution here, in using the word “suggest”. It is the case that most of these reviews, as well as cross-national epidemiological studies [36], found both depression and anxiety to be more prevalent in the Western world than in less developed regions such as South Asia. While genetic, sociocultural, environmental and other factors might contribute to real differences, there are important methodological factors to consider that influence prevalence estimates. Commonly these relate to sampling methods, but of specific concern here are the instruments used. Most Western studies utilized diagnostic interviews, while surveys in the less-affluent world used symptom-based scales to screen for psychiatric caseness. Accordingly, HADS, which we used, is a screening instrument for estimating the point prevalences of anxiety and depression, and as such it has limitations. It detects the subjective manifestations of anxiety and depression [24], while vegetative or somatic symptoms of distress forming parts of the diagnostic classifications (DSM [37] or ICD [38]) may not be sufficiently captured. Surveys dependent on HADS and similar instruments may therefore *underestimate* actual prevalences, as has been discussed both in the review on CMDs [5] and in a WMH survey from China [39].

The Chinese study [39] emphasized the relevance of sociocultural protective factors (family structure, neighbourhood), which are believed to play a buffering role in most Asian countries [5], including Nepal, against the distress associated with anxiety or depression. In a different vein, stigma associated with the widespread belief that disclosure of mental illness might lead to embarrassment and discrimination is more common in underdeveloped societies, and may contribute towards underreporting of mental as opposed to physical symptoms; this too was evidenced in a WMH multicentre study [40]. Because these issues were likely to apply to our study, our findings of excessively prevalent depression and anxiety in Nepal appear even more remarkable since they were unlikely to be overestimates.

From the public-health perspective, the importance of their very high prevalences lies in the associations of both depression and anxiety with substantial disability. There is a wealth of evidence of this, including the data from GBD 2013 [4]: globally, major depressive disorders are the 2^nd^ highest cause of YLDs (51.8 million per year), and dysthymia, which is also expected to be captured by HADS-Depression, is 16^th^ (another 9.8 million YLDs per year); anxiety disorders are 9^th^ (24.4 million YLDs per year). These GBD estimates are based on the global mean prevalences—well below those we have found in Nepal. In other words, the disability these disorders give rise to globally [4], great though it is, may not at population level match that in Nepal. Important also are our findings that all of HADS-A, HADS-D and HADS-cAD were associated with low QoL and high neuroticism, illustrations of their major effects on functioning at individual level.

With regard to associations, damage to family or social functioning is linked to mental ill health [41]. As evidence of this, we found HADS-cAD to be more prevalent among widows. Similarly, a cross-national survey [36], as well as two Asian studies—one from Iran [16] and one from China [42]—showed a high prevalence of CMDs among the widowed. Beyond the stress precipitated by a major family life event, widowhood entails substantial deviance in the societal role as well as in self-perception: widows perceive a lack of social support compared with those who are married [43].

Society is made up of households that are characteristic of the habitation where they stand. We found substantial associations between mental health and the location of the home: HADS-A and HADS-cAD were significantly more prevalent among the urban population, as was seen for the anxiety disorders in one of the global reviews [34]. Similarly, in India [44], multiple effects of unplanned urbanization including fast population growth, environmental degradation and sociocultural conflicts were cited as possible contributors of escalating mental-health problems, particularly depression and anxiety, among city populations. These may be applicable also in Nepal: decade-long political conflicts resulted in rapid migration of villagers into nearby cities, thus swiftly expanding the urban population [45]. In addition, other factors may lead to an increase in the prevalence of psychiatric disorders in the cities [46]: for example, the tendency of some mentally ill people to settle in towns rather than in the countryside, possibly to protect them from social stigma, to be away from the difficulties of rural life, to obtain proper care from social welfare institutions or better treatment, in search of jobs, or just to beg.

Habitation in Nepal also includes high hills and mountains, which cover almost one third of the total land area of the country [47]. So far, no study has explored psychiatric illnesses among the occupants of these territories. This was the first research in the South Asian Region to demonstrate the possible effect of geographical elevation on mental health. It showed HADS-cAD to be more prevalent above 2000 m. Two studies in Peru [48, 49] and a US study [50] suggested the role of hypoxia and mitochondrial dysfunction as the possible link between altitude and depression. There are also studies on high-altitude ascenders from China [51] and among porters and trekkers in Nepal [52] that found anxiety to be one of the most recorded medical symptoms. But further work is necessary to elucidate whether biological conditions or psychosocial factors related to life adversity, isolation or the limited access to mental-health facilities in these areas are responsible for the mental-health problems.

Our findings of a negative association between alcohol use and HADS-A (as well as HADS-cAD), and of a positive relationship between marijuana use and HADS-D, need cautious interpretation. It is not so simple to capture the impact of alcohol use in a country like Nepal where drinking is considered an integral part of social functioning in most of the so-called *Matwali* community, which traditionally is prone to drinking [53]. Similarly, marijuana, which is also culturally accepted among the Nepalese, especially during certain religious functions [54], may not show its true relationship with mental health. We were unable fully to evaluate the potential relationships between substance dependence and mental health because the length of the questionnaire restricted us from adding more questions. Further studies are necessary in this area.

Contrary to global [5, 33, 34] and local studies [10, 15, 16], we found no gender associations. Perhaps the somatic symptoms that generally are reported more frequently by depressed females [55] were not sufficiently captured by HADS, which was originally constructed to detect emotional symptoms among hospital populations, and therefore pays less attention to bodily symptoms [24].

Although many studies have shown associations between poverty and CMDs in the LAMI countries [56], we did not find direct support for these. It may be that our questions on annual household consumption and monthly expenditure did not estimate well the socioeconomic status of the household; the responses may have been vaguely reported by the participants, many of whom were illiterate [30]. In Nepal, where people generally are poor [19], it is challenging to find a suitable measure of socioeconomic status in relation to CMD caseness. Income is not a reliable indicator, consumption cannot be assessed using direct monetary measures and proxy measures such as educational status have limited usefulness in this country with so much illiteracy; all these issues have been discussed in an earlier publication [20]*.* Nevertheless, our observation of high prevalence of both anxiety and depression among the Nepalese with relatively low socioeconomic status may well establish the link between poverty and mental ill-health. As concluded in a WMH survey, income inequality is a possible factor promoting chronic illnesses like depression [36], but more so in high-income countries than in the LAMI countries.

Our study was built upon tried and tested methodology [20], a large sample size, a very high participation rate achieved through careful sampling methodology [21], completeness of data and representativeness of the population’s geographical and cultural diversities. These were the strengths of this study. The cross-sectional nature of the study obviously could not capture the longitudinal, relapsing and remitting course of depression and anxiety, or illustrate the temporal direction of associations with sociodemographic factors [36]. The limitations in the use of HADS rather than expert clinical interview have been discussed above. However, these are considerably offset: we believe culturally validated study instruments account for socially acceptable outcomes better than the ethnically insensitive diagnostic classifications relied upon in various cross-national [35] and global reviews [33].

## Conclusion

Our study has opened a neglected research arena [18]. As a pioneering work in assessing prevalences of the most common mental disorders nationwide, it also brings out important implications for advancing the field of mental health in Nepal. We found that depression and anxiety were highly, perhaps excessively, prevalent in the country, while noting that these disorders are globally the second and ninth highest causes of disability. Also, we found that disadvantaged groups such as widows and those tolerating hardships and scarcity in the high hills were more affected. From the public-health perspective, these are clear, compelling and urgent messages. Depression and anxiety must be among the health-care priorities in Nepal; there must be expansion, and wider delivery, of mental health care in the country. In view of the scarce resources, limited health budget and lack of specialized psychiatric services, integrating care for a substantial majority of those affected by these disorders into the fabric of national primary health-care services would be a reasonable goal.

### Ethics approval and consent to participate

The study protocol was approved by the Nepal Health Research Council (NHRC), the Institutional Review Committee of Kathmandu University School of Medical Sciences, Dhulikhel Hospital (IRC-KUSMS), and The Central Regional Committee for Health and Research Ethics in Norway. Informed consent was given by all participants and confirmed either by signature or by fingerprint.

### Consent for publication

Prior to the interview, all prospective participants were given written information approved by the ethics committees, which described in Nepali the nature and purpose of the study and the implications of taking part. This information clearly stated that consent to participation included consent to publication by the researchers of participants’ anonymized data, which would be held at the Norwegian University of Science and Technology. Prospective participants who were literate read this information. To those who were illiterate, the interviewers read the information in the presence of family members.

### Availability of data and materials

The data contributing to these analyses are held on a secure database at Norwegian University of Science and Technology (NTNU) in accordance with European data-protection legislation and consents given by participants. Researchers or clinicians seeking access to these data for academic non-commercial purposes are welcome to submit a request to the corresponding author (AR). All such requests will be met whenever possible.

## Abbreviations

AOR: adjusted odds ratio
BP: blood pressure
BMI: body mass index
cAD: comorbid anxiety and depression
CI: confidence interval
CMD: common mental disorder
DSM: diagnostic and statistical manual of mental disorders
EPQRS-N: eysenck personality questionnaire revised short form-neuroticism
GBD: Global Burden of Disease
HADS: Hospital Anxiety and Depression Scale
HARDSHIP: headache-attributed restriction, disability, social handicap and impaired participation
ICD: International Classification of Diseases
JNC: Joint National Committee
LAMI: low-and-middle-income
NHRC: Nepal Health Research Council
NTNU: Norwegian University of Science and Technology
OR: odds ratio
QoL: quality of life
SPSS: statistical package for social science
WHOQOL-8: World Health Organization Quality-of-Life 8-question scale
WMH: World Mental Health
YLD: year of life lost to disability
